# Designing Learning Intervention Studies: Identifiability of Heterogeneous Hidden Markov Models

**DOI:** 10.1017/psy.2025.10024

**Published:** 2025-07-22

**Authors:** Ying Liu, Steven Culpepper

**Affiliations:** Department of Statistics, University of Illinois Urbana-Champaign, Champaign, IL, USA

**Keywords:** cognitive diagnosis model, heterogeneous hidden Markov models, identifiability, restricted latent class models

## Abstract

Hidden Markov models (HMMs) are popular for modeling complex, longitudinal data. Existing identifiability theory for conventional HMMs assume emission probabilities are constant over time and the Markov chain governing transitions among the hidden states is irreducible, which are assumptions that may not be applicable in all educational and psychological research settings. We generalize existing conditions on homogeneous HMMs by considering heterogeneous HMMs with time-varying emission probabilities and the potential for absorbing states. Researchers are investigating a family of models known as restricted HMMs (RHMMs), which combine HMMs and restricted latent class models (RLCMs) to provide fine-grained classification of educationally and psychologically relevant attribute profiles over time. These RHMMs leverage the benefits of RLCMs and HMMs to understand changes in attribute profiles within longitudinal designs. The identifiability of RHMM parameters is a critical issue for ensuring successful applications and accurate statistical inference regarding factors that impact outcomes in intervention studies. We establish identifiability conditions for RHMMs. The new identifiability conditions for heterogeneous HMMs and RHMMs provide researchers insights for designing interventions. We discuss different types of assessment designs and the implications for practice. We present an application of a heterogeneous HMM to daily measures of positive and negative affect.

## 1 Introduction

The increasing availability of data from online learning systems, wearables, and handheld devices provides researchers with new opportunities to track development, evaluate interventions, and recommend content. These novel data structures often arrive as a multivariate time series that require methods to diagnose and classify respondent outcomes in a longitudinal fashion. Hidden Markov models (HMMs) provide a general framework for understanding changes in latent states over time. HMMs consist of two components: (1) an emission matrix 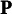 that describes the distribution of observed responses given latent states; and (2) a transition matrix 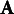 that governs the likelihood of transitioning between states over two adjacent time points. HMMs are designed to relate an observed 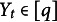 with a hidden state 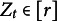 for 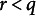 where we use the notation 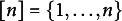 to denote the set of natural numbers from 1 to 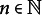 . Recent research (e.g., see Kaya & Leite, 2017; Li et al., 2016; Madison & Bradshaw, 2018a; Madison et al., 2024; Zhan et al., 2019; Zhang & Chang, 2020) adapted the HMM framework for the diagnostic context with a class of models we refer to as restricted HMMs (RHMMs), which map 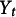 onto a more parsimonious collection of binary attributes, 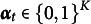 . RHMMs combine features of cognitive diagnosis models (CDMs; e.g., see de la Torre & Douglas, 2004; Junker & Sijtsma, 2001; Roussos et al., 2007; Stout, 2002) and HMMs. Specifically, RHMMs define 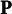 according to a CDM to classify respondent states at a given time as well as a first-order HMM to describe changes in latent states over time.

The combination of the classical HMM framework with CDMs has the potential to advance learning research and to identify interventions that accelerate learning (Ye et al., 2016). It is therefore critical to understand technical details concerning the suitability of the HMM and RHMM frameworks for educational and psychological research. There is extensive research exploring the necessary and sufficient conditions for deploying CDMs (e.g., see Chen et al., 2015, 2020; Gu & Xu, 2021; Liu et al., 2013; Xu, 2017; Xu & Shang, 2018) and several studies are dedicated to understanding the identifiability of HMMs (Allman et al., 2009; Bonhomme et al., 2016), but there is less research available to guide applications that combine CDMs and HMMs (Liu et al., 2023). RHMMs have features of both CDMs and HMMs, so it is important to understand the assumptions of both components. Classical HMMs assume constant emission and transition probabilities over time in addition to positive long-run, stationary probabilities of residing in every state. The focus of this article is on relaxing the constant 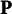 and positive long-run probability assumptions, so we consider a constant transition matrix, 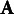 , throughout the remainder of this manuscript. It is important to understand the practical implications of these two assumptions. The first assumption that 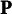 is constant over time implies that: (1) the support of the observed indicators, 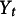 , and hidden states, 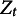 , does not change over time; and (2) the observed 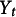 are parallel measurements in the sense that 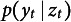 is constant across *t*. Accordingly, the constant 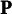 assumption could be reasonable whenever researchers observe a common set of measurements over time, such as settings that monitor health and well-being and public opinion with a fixed instrument. However, there are no guarantees the constant 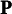 assumption is feasible in all settings especially when the items change over time.

The second assumption deals with the stationary (long-run) probability of residing in the hidden states, which is denoted by 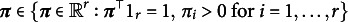 (i.e., the support for 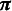 is the *r*-dimensional simplex). The second assumption requires that 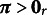 where 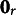 is an *r*-vector of zeros and “ 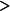 ” is interpreted as an element-wise inequality. We can write 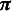 as a limit involving the initial distribution and the transition matrix. Specifically, the initial distribution is 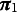 where element *j* corresponds with 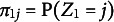 . Iterating forward we find the distribution for time 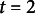 , 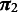 , to have elements, 
(1)

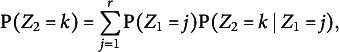

which is more simply stated as 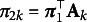 where 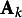 is the *k*th column of 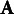 . Consequently, 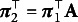 and iterating forward to time *n* implies that the distribution of 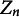 is 
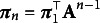
 . The long-run distribution is 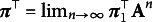 . The extent to which 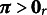 is related to whether the Markov chain is irreducible. A Markov chain is irreducible if it is possible to move from a given state *j* to every other state *k* in finite time. The irreducibility assumption is violated (and 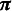 includes elements equal to 0) whenever 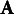 includes absorbing states. For instance, state *k* is an absorbing state if 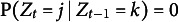 for all *j*. The irreducibility assumption is satisfied in many content areas, but we can expect it to be violated in educational studies that advance learning. That is, interventions are designed to facilitate skill development toward the state of mastery, 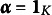 , which is an absorbing state whenever we believe it is unlikely to unlearn skills. Whereas forgetting skills may occur over a longer horizon (e.g., summer slide), the irreducibility assumption is generally untenable within shorter windows defined by typical intervention studies (e.g., days, weeks, or months).

The union of CDMs and HMMs offers a powerful framework for providing fine-grained diagnostic classifications, but care is needed to ensure foundational assumptions are consistent with application domains. RHMMs have been applied to track changes in spatial rotation skills (Chen et al., 2018; Wang et al., 2018; Yigit & Douglas, 2021), introductory probability (Liu et al., 2023), middle school mathematics (Li et al., 2016), digital literacy (Liang et al., 2023), a cluster-randomized controlled trial of mathematics for students with disabilities (Madison & Bradshaw, 2018b), and geometric sequences (Chen & Culpepper, 2020). An important observation is that many applications have deployed RHMMs that relax the constant 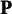 and 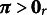 assumptions. For instance, prior research applied more general RHMMs that include: (1) time-varying emission probabilities with 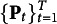 ; and (2) the studies were applied in educational contexts with transition matrices that include absorbing states. Consequently, applied RHMM research has outpaced current theory related to the identifiability RHMM parameters.

RHMMs provide a useful framework for educational and psychological research. However, despite the widespread application and investigation of RHMMs, there is less research available regarding how to design intervention studies to ensure RHMM parameters are identified. Model identifiability is a critical issue for statistical inference. In the context of intervention studies, researchers need assurance that model parameters are identified in order to make claims about the effect of interventions on development.

Prior research established conditions for identifying parameters of HMMs to guide the design of diagnostic interventions. Several studies explored identifiability conditions for the case of continuous responses where 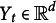 for 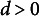 (Gassiat et al., 2016; Gassiat et al., 2020; Gassiat & Rousseau, 2016). We consider discrete responses, so the identifiability conditions derived under the assumption of continuous responses are not applicable for our case. There are several papers that explored the identifiability of unrestricted HMMs with constant emission and transition matrices and an irreducible transition matrix for discrete responses (Allman et al., 2009; Bonhomme et al., 2016; Cole, 2019; David et al., 2024; Tune et al., 2013). Specifically, Cole (2019) describes a log-likelihood profile method for establishing local identifiability of HMMs as well as a symbolic algebra strategy for establishing global identifiability of HMMs. One limitation of Cole (2019) is the fact that “...for most HMMs the exhaustive summary will be symbolically too complex for a symbolic algebra package to solve the appropriate equations” (p. 117). A paper from the theoretical statistics literature (Bonhomme et al., 2016) showed how to establish strict identifiability conditions for HMMs using results pertaining to the simultaneous diagonalization problem. Specifically, Bonhomme et al. (2016) showed that HMMs are strictly identified whenever 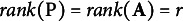 . Allman et al. (2009) proved that HMMs are generically identifiable, which means that the non-identifiable parameter values reside in a measure zero set of the larger parameter space, provided that researchers observe enough time points relative to the number of observed response patterns and the number of hidden states. Tune et al. (2013) consider an extension of Allman et al. (2009) by studying the identifiability of HMMs for a multiple observer model, which in the context of psychometrics corresponds with a model that includes several items at each time point. David et al. (2024) considers the identifiability of HMMs when external signals, such as observed time-varying covariates, are available as predictors of both the hidden 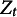 and 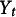 . David et al. (2024) show that when external signals are available that it is possible to establish conditions for identifying parameters of HMMs with time-varying emission and transition matrices. Recently, Liu et al. (2023) established identifiability conditions for RHMMs and showed that identification is closely linked with properties of the emission and transition matrices.

Existing research provides helpful guidelines for designing diagnostic intervention studies, but the studies are limited by several critical assumptions. First, the five studies (Allman et al., 2009; Bonhomme et al., 2016; Cole, 2019; Liu et al., 2023; Tune et al., 2013) assume constant emission and transition matrices over time in addition to an irreducible transition process (i.e., 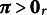 ). In contrast, we present new conditions for identifying HMMs and RHMMs with heterogeneous 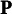 and we allow for the possibility of absorbing states. Although the David et al. (2024) relaxes the assumption of constant 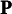 and 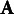 matrices, their identifiability conditions assume the availability of external covariates that relate to both hidden states and observed responses. In the context of psychometrics, David et al., 2024’s requirement for external signals to relate to both hidden states and observed responses is analogous to educational and psychological researchers needing test-taker covariates that contribute to differential item functioning by relating to both the measurement and transition models. The novel theorems in this article provide identifiability conditions without the need for external signals. Furthermore, David et al. (2024) assumes a stationary process for the observed and hidden states. Consequently, David et al. (2024) is not directly applicable in educational contexts as it requires external signals and assumes a stationary process, which does not accommodate learning and absorbing states.

The purpose of this paper is to fill the theory–practice gap by offering new insights about the identifiability of heterogeneous HMMs and RHMMs when 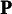 is time-varying and 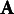 includes absorbing states. We therefore consider the case where the emission matrix changes with time (i.e., a time-varying model). Furthermore, we discuss conditions for cases where the Markov chain both satisfies and does not satisfy the irreducibility assumption. The remainder of this paper includes five sections. The first section discusses identifiability conditions for an unrestricted heterogeneous HMM and the second section focuses on the case of RHMMs. The third section discusses the implications of the new identifiability conditions in terms of practical considerations for assessment design. The fourth section presents a Bayesian approach for estimating the model parameters of the heterogeneous HMM, a simulation study to demonstrate parameter recovery, and an application to a dataset concerning respondents daily changes in positive and negative affect. The final section discusses the implications of the results and directions for future research. Note that we include all proofs and technical details in the appendix.

## 2 Unrestricted heterogeneous HMMs

We begin our discussion by focusing on the finite heterogeneous HMM framework where the emission probabilities vary over time. The first subsection presents an overview of HMMs and the second subsection delves further into the identifiability of heterogeneous HMMs with time-varying emission matrices and includes two new theorems.

### 2.1 Overview

**Figure 1:**
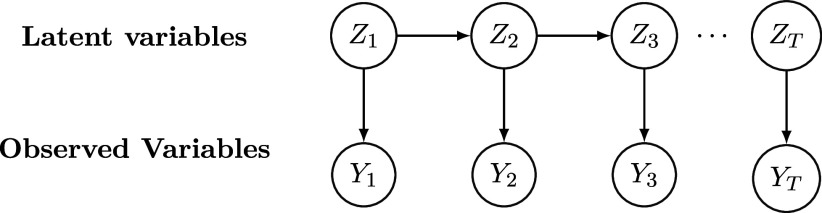
First-order hidden Markov model (HMM) with latent 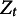 underlying the observed 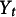 variables.

Consider an irreducible, aperiodic stationary Markov chain 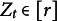 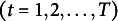 , with a time-invariant 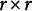 transition matrix 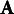 , and a stationary distribution 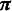 such that 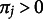 and 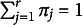 . We assume the the observed 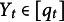 are conditionally independent given the hidden states. That is, we assume 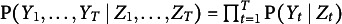 . Let 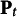 denote the time-varying emission matrix with dimension 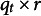 and 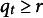 , which contains the conditional probabilities 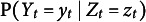 , where the column entries are indexed by 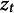 and rows correspond to observation patterns 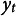 . We consider a first-order Markov model for the latent 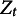 ’s, which assumes that given 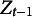 , 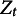 is conditionally independent of past values for the hidden states, 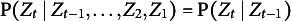 . The probability of transitioning between states over time is governed by the transition matrix 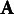 . That is, the probability of transitioning from state 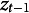 at time 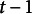 to state 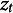 at time *t* is 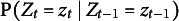 , which corresponds with row 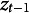 and column 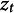 of 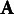 . Figure 1 presents a graph of an HMM where the transition matrix governs the relationship among the hidden states and the observations are conditionally independent given the 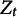 ’s.

### 2.2 Identifiability

We next discuss the identifiability of heterogeneous HMMs. We first discuss conditions for establishing strict identifiability to ensure a unique mapping between the parameter space and likelihood function. We conclude this subsection by presenting weaker generic conditions that are needed for the non-identifiable parameter values to reside within a measure zero set.

#### 2.2.1 Strict identifiability

Identifiability is an important prerequisite of statistical parameter inference. A model is identifiable if different values of model parameters corresponds to different probability distributions of observed variables. Model identifiability ensures that its underlying parameters can be consistently estimated.

Let 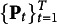 denote the *T* emission matrices. In the context of HMMs, we denote the parameter space of 
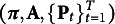
 by 
(2)

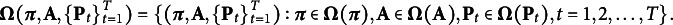

The parameter space for the stationary distribution is 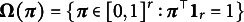 . The parameter spaces for 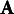 and 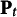 are 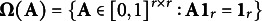 and 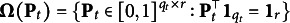 .

Definition 1 **(Strict identifiability).**

The parameters 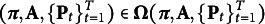 are identifiable when 

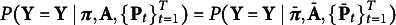




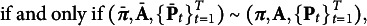

where 
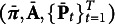
 is another value from the parameter space 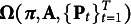 and “ 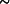 ” means two parameter values are equivalent up to a permutation of hidden states.

The conditions shown in Assumption 1 are needed to establish the strict identifiability condition in Theorem 1.

Assumption 1. Suppose for 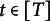 , 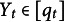 follows an HMM with hidden state 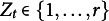 , time-specific 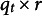 emission matrix 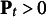 , time-invariant transition matrix, 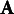 , and stationary distribution with 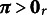 and 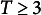 with parameters that satisfy

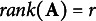
 ;

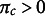
 for all 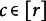 ;

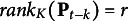
 , 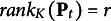 and 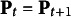 for some 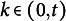 and 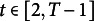 .

Remark 1. Assumption 1b requires a positive stationary distribution and excludes the possibility of absorbing states. Assumption 1c involves a condition on the Kruskal rank of certain emission matrices. The Kruskal rank of a matrix with *r* columns equals *r* if and only if the matrix has full column rank. See Definition 13 in the Appendix A for additional details about the Kruskal rank.

We next present our Theorem related to identifiability of heterogeneous HMMs.

Theorem 1 **(Strict identifiability for heterogeneous HMMs).**

Under Assumption 1, then 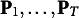 , 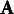 , and 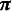 are identified, up to label-switching.

Proof can be found in Appendix A.

Remark 2. Theorem 1 still holds if we have a slightly different condition 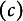 in Assumption 1 where 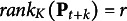 , 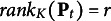 , and 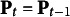 for some 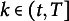 and 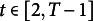 .

#### 2.2.2 Generic identifiability

Theorem 1 established strict identifiability conditions for heterogeneous HMMs, which could be too strong for practical data analysis. A weaker notion of identifiability is referred to as generic identifiabilty, which was first introduced in Allman et al. (2009). Generic identifiability permits the presence of certain exceptional parameter values for which strict identifiability does not apply; however, these non-identifiable parameters must form a set with Lebesgue measure zero. Because the non-identifiable parameters are confined to a measure zero set, one is unlikely to face identifiability problems in performing inference. Therefore, generic identifiability is generally adequate for data analysis purposes. For example, Allman et al. (2009) demonstrated that generic identifiability necessitates fewer consecutive observed variables to fully determine the distribution of an HMM compared to strict identifiability. We next discuss generic identifiability of heterogeneous HMMs.

Let 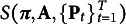 denote the set of non-identifiable parameters from 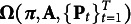 : 
(3)

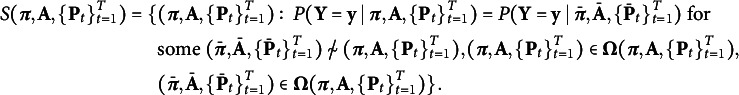

Definition 2 **(Generic Identifiability).**

The parameter space 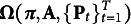 is generically identifiable, if the Lebesgue measure of 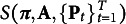 with respect to parameter space 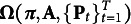 is zero.

Theorem 2 **(Generic Identifiability for Heterogeneous HMMs).**

For a heterogeneous HMM, the parameter space 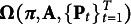 is generically identifiable up to label-switching if 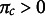 for all 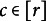 , and

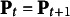
 for 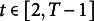 ;

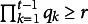
 , 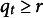 , 
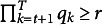
 .

Proof is shown in Appendix B. The generic condition for heterogeneous HMMs is weaker than the strict condition. Specifically, Theorem 2 imposes conditions on the dimensions of the emission matrices and does not require that any particular emission matrix is full rank.

## 3 Heterogeneous restricted HMMs (RHMMs)

The previous section discussed identifiability of heterogeneous HMMs where the observed response and hidden states are categorical random variables, the emission matrix 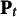 is unrestricted, and the transition matrix 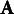 corresponds with an irreducible and aperiodic first-order Markov process with stationary distribution 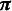 . The purpose of this section is to introduce new identifiability conditions for restricted HMMs where restrictions are imposed upon the emission matrix. Accordingly, this section includes three subsections. The first subsection includes a discussion that connects restrictions in the emission matrix with popular diagnostic models. The second and third subsections focus on the identifiability of RHMMs for two types of models depending upon whether the transition matrix corresponds with an irreducible process. Accordingly, the second subsection corresponds with the case where 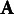 is irreducible with stationary distribution 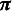 and the third subsection presents identifiability conditions for the case where 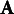 includes absorbing states.

### 3.1 Overview and definitions

This section considers the identifiability of the heterogeneous version of RHMMs, which include a binary vector of latent attributes, 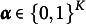 as opposed to the 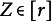 in the previous section. The hidden state for RHMMs at a given time *t* consists of a binary profile 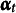 and indicates whether a given respondent possesses one of the 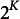 possible profiles for the *K* attributes. We next define a general model for a categorical response 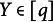 .

Definition 3 **(Nominal diagnostic model; NDM).**

A general model for a categorical response 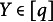 is the NDM, which has an item response function of: 
(4)

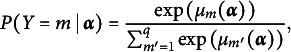

where for 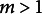 , 
(5)

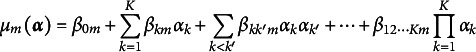

includes the main-effects and interaction-effects among the attributes. Note that the parameters for the 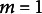 case satisfies 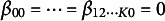 to identify the model.

Definition 4 **(Structure matrix).**

Let 
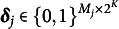
 be a binary matrix such that the element in the *m*th row and *p*th column 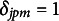 if 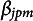 is active and non-zero and 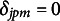 if 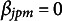 and inactive.

Remark 3. The NDM is designed for nominal data and there are numerous special cases for dichotomous and polytomous response data. We refer readers to de la Torre & Douglas (2004) for a review of popular dichotomous diagnostic models. For example, Chen et al. (2020) provide an overview of how different configurations 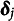 corresponds with different diagnostic models for the 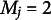 case. Additionally, there are several studies on the development of polytomous diagnostic models (e.g., see Culpepper, 2019; Fang et al., 2019; Ma & de la Torre, 2016).

An important feature for RHMMs is that we typically observe responses to multiple items at a given point in time.

Definition 5 **(Multi-item emission matrix).**

Suppose there are *J* total items. We let 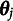 be the 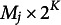 matrix of response probabilities such that element 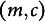 denotes the probability of observing a response of *m* on item *j* for members of class *c*. We let 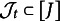 denote the subset of items administered at time *t*. Notice that the definition of 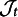 allows for the possibility that different items are administered over time. Accordingly, under the assumption that the 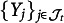 ’s are conditionally independent given 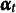 the emission matrix for a given time *t* is 
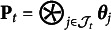
 where 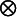 is a Khatri–Rao product of matrices (see Definition 8 in Appendix A).

We next present an example to demonstrate the previously defined components of the NDM.

Example 1. Suppose 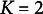 and 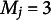 , so 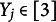 and the 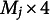 matrix of response probabilities, 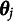 , is, 
(6)

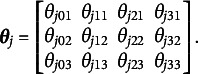

The 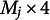 matrix of regression coefficients is 
(7)

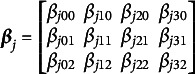

and the corresponding 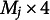 structure matrix 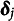 is 
(8)

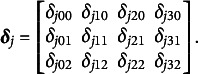

Note that 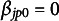 and 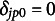 for all *p* to identify the model parameters and it is customary to include intercepts in the model, which is imposed by the restrictions 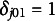 and 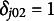 .

### Irreducible process with stationary distribution 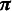


3.2

The purpose of this subsection is to establish the identifiability of RHMMs with an irreducible and aperiodic transition matrix 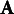 . We first discuss conditions for strict identifiability and then conclude this subsection with results for generic identifiability.

It is important to note that there is a one-to-one mapping between the 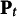 ’s and the NDM 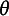 and 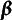 parameters. Therefore, RHMM parameters based upon the NDM are strictly identified whenever the conditions in Assumption 1 for Theorem 1 are satisfied. However, verifying strict identifiability of the NDM can be more cumbersome given there are many parameters and ways to construct the 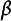 ’s so that the formed emission matrices have full column rank. Consequently, we first discuss conditions for strictly identifying RHMM parameters for the special case of binary RHMMs prior to focusing on generic identifiability for the more general setting.

Example 2 **(Binary RHMMs).**

Suppose 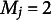 for all *j*, so that 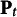 is a 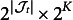 matrix and 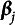 and 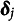 are 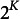 -vectors. The structure matrix in this setting for the items administered at time *t* is 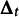 is a 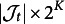 binary matrix with rows defined by the associated 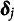 for 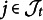 . Accordingly, 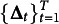 are the structure matrices for the items forming 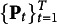 .

Assumption 2. Consider the conditions for the structure matrix, 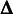 , of binary RHMMs:

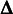
 takes the form 
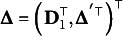
 after row swapping, where 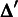 is any 
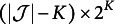
 binary matrix and 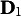 takes the form 

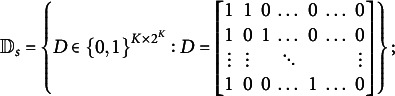

- For two classes of respondents, there exists at least one item in 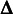 , in which they have different success probabilities.

Remark 4. Note that we use the convention that the first column of 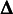 corresponds to the intercept, the next *K* columns the main-effects for the attributes, the next 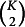 columns the two-way interaction effects, etc. The last column corresponds with the *K*-way interaction effect.

Corollary 1 **(Strict identifiability of Binary RHMMs).**

Any parameter from 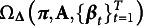 is strictly identifiable up to label-switching, if:

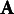
 satisfies Assumption (1a);

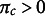
 for all *c*;

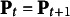
 for some 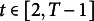 ; and

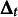
 and 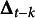 satisfy Assumption (2a) for 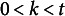 .

Proof is shown in Appendix C.

Remark 5. Corollary 1 requires *K* simple structure items are administered in 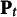 and 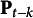 . A weaker condition is available by imposing restrictions on pairs of items as described by Culpepper (2023).

Assumption 3. Consider the conditions for the structure matrix, 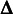 , of binary RHMMs:

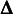
 takes the form 
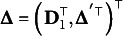
 after row swapping, where 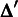 is a 
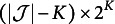
 binary matrix and 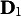 is of the following form: 

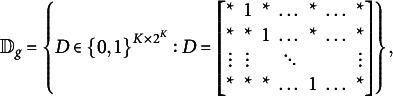

where 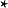 can be either 0 or 1.
- In 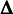 , each main-effect must appear at least once. There exists a *j* such that 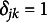 for any 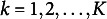 .

Corollary 2 (Generic identifiability for binary RHMMs with 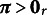 ).

The parameter space 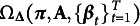 is generically identifiable up to label-switching, if:

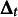
 and 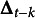 for 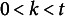 satisfy Assumption (3a); and

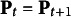
 for some 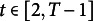 .

Proof can be found in Appendix D.

### 3.3 Identifiability for absorbing-state, multi-item HMM

The previously discussed classical HMM assumes that 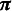 is the long-run stationary distribution with the requirement that all elements are non-zero, 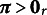 . The requirement of non-zero elements of 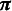 implies the absence of an absorbing state, which is inconsistent with the goals of education. That is, learning interventions are designed to transition students into states with greater skills, knowledge, and abilities. The purpose of this subsection is twofold. First, we present results for identifying parameters of multi-item HMMs for the case where 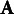 includes absorbing states. Second, we present Corollaries that are specific to the RHMM setting. We establish identifiability using Kruskal’s theorem for the uniqueness of three-way arrays.

Suppose for 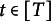 , 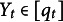 follows a multi-item HMM with hidden state 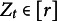 , time-specific 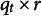 emission matrices 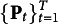 , time-invariant transition matrix, 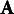 , and an initial distribution with 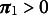 , two or more time points (i.e., 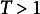 ) with 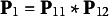 where 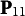 and 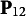 are column-wise stochastic matrices and 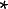 denotes a Khatri–Rao product, which is a column-wise Kronecker product (see Definition 8 in Appendix A for more details).

Remark 6. An important component of the absorbing state HMM is that the emission matrix for the first time point can be written as a Khatri–Rao product of two emission matrices. This assumption implies that there are two responses at time 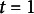 with 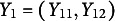 such that 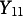 and 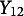 are conditionally independent given 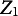 .

A first step for using Kruskal’s theorem to establish parameters of the absorbing state RHMM are identified is to write the model as a three-way array. Specifically, we condition the observed responses on 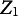 , 
(9)

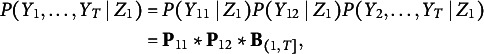

where 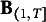 is the 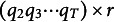 emission matrix for 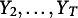 given 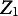 (see Definition 9 and Equation A4 in Appendix A for more details). We write the marginal distribution, 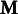 , of 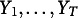 in the three-way array representation as 
(10)

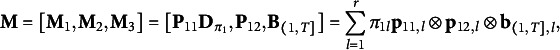

where 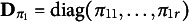 , 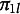 is the *l*th element of the initial distribution 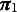 , and 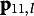 , 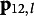 , and 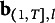 are the *l*th column of 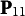 , 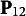 , and 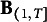 , respectively.

Theorem 3 **(Strict identifiability for heterogeneous, multi-Item HMM with absorbing states).**

The parameters 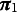 , 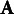 , and 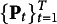 of a multi-Item HMM with absorbing states are strictly identifiable up to label-switching if:

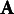
 satisfies Assumption (1a);

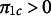
 for all *c*;

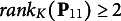
 , 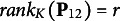 , and 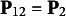 .

Proof is shown in Appendix E.

We reparameterize the multi-item HMM as a RHMM by replacing 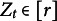 with the latent attribute profile 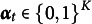 .

Example 3 **(Binary RHMMs with Absorbing States).**

Suppose 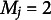 for all *j*, so that 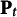 is a 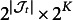 matrix and 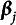 and 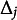 are 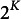 -vectors. 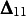 , 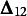 , 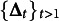 are the structure matrices for the items forming 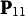 , 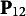 , and 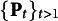 , respectively.

We next apply Theorem 3 to establish a corollary for the identifiability of binary RHMMs.

Corollary 3 **(Strict identifiability of binary RHMMs with absorbing states).**

Any parameter from 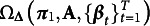 is strictly identifiable up to label-switching if:

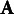
 satisfies Assumption (1a);

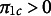
 for all *c*;

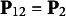
 ; and

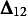
 satisfies Assumption (2a), and 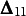 satisfies Assumption (2b).

Proof can be found in Appendix F.

Remark 7. Corollary 3 relies upon Assumption (2a), which requires that *K* simple structure items are administered in 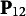 . Note that a version of Corollary 3 is possible such that strict identifiability can be achieved with weaker conditions imposed upon on pairs of items, or dyad (e.g., see Culpepper, 2023).

Corollary 4 **(Generic identifiability for binary RHMMs with absorbing states).**

The parameter space 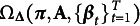 is generically identifiable up to label-switching, if:

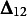
 satisfies Assumption (3a) and 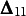 satisfies (3b); and

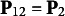
 for some 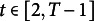 .

Proof can be found in Appendix G.

Remark 8. Corollaries 1, 2, 3, and 4 are focused on the case of binary response data, but these results can be easily extended to more general response models using existing results. For instance, we can extend Corollaries 1 and 3 to the case of nominal response RHMMs by replacing condition (4) with the conditions for the 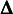 structure in Liu and Culpepper (2024 Theorem 1). Moreover, we can establish generic identifiability for a nominal response RHMM by replacing (1) in Corollaries 2 and 4 with the conditions for the 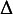 structure in Liu and Culpepper (2024, Theorem 2).

## 4 Practical considerations for assessment design

**Figure 2:**
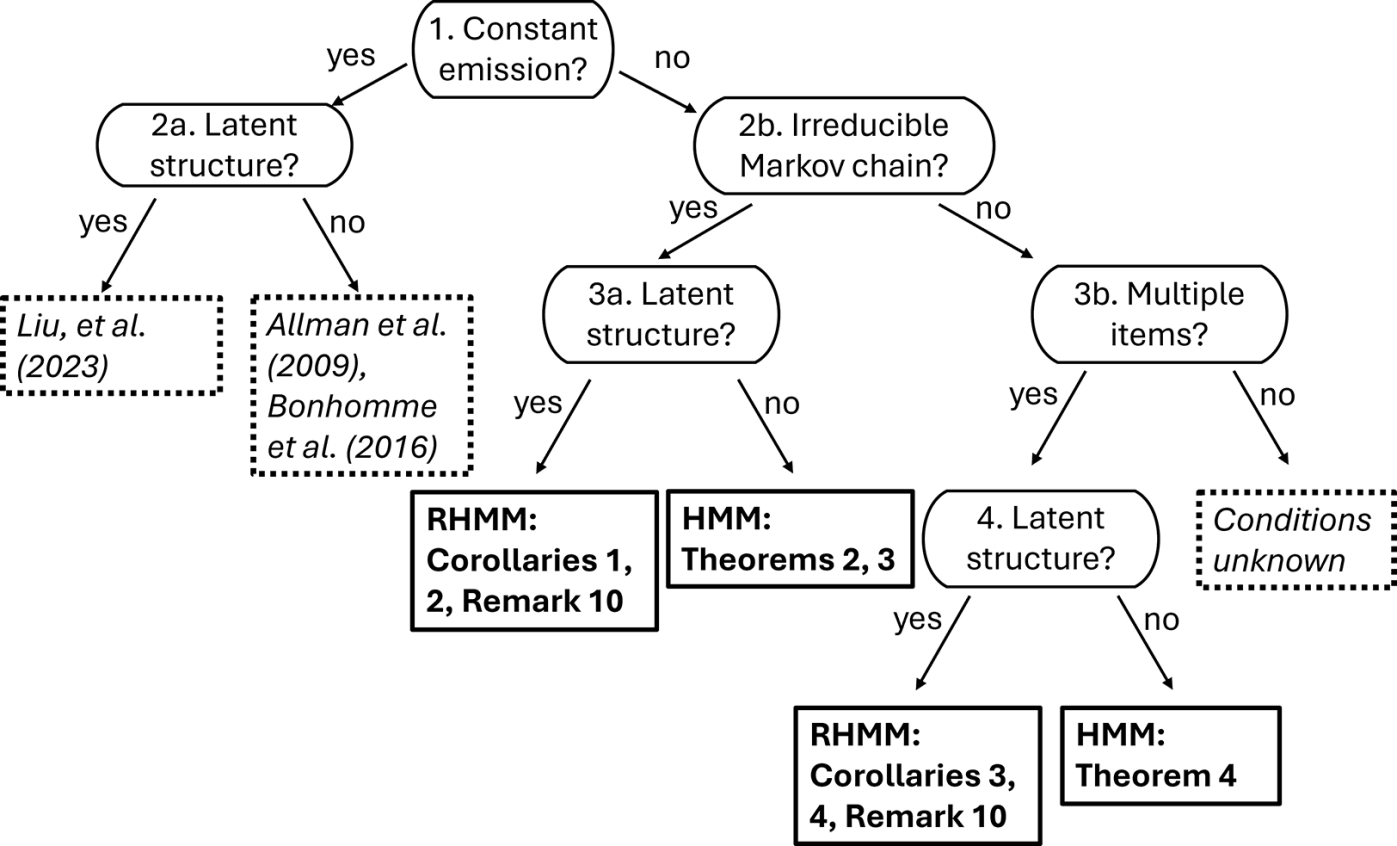
Flowchart of identifiability results for different types of hidden Markov models. *Note*: The boxes with dotted borders and italicized text indicate conditions from existing research whereas the bold boxes indicate conditions established in this paper.

The purpose of this section is to provide a summary of the aforementioned results for practitioners who design assessments within the HMM or RHMM frameworks. Figure 2 includes a flowchart of the relevant decision points for deciding upon which results to use as a guide for designing studies.

The flowchart includes four layers of decision points. The first decision at the top of the flowchart requires practitioners to decide whether the emission matrix will be constant over time. An important factor for determining whether 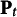 changes with time is the extent to which researchers can administer an instrument over time that has invariant response probabilities. The instrument for an invariant 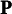 may manifest differently for various contexts. In the case of mental health assessment, the instrument might include one or more standard items regarding patients mood or mental state. In the context of education, the instrument could consist of a collection of common items or parallel items, which require mastery of the same mental processes. If a parallel item design is deployed it is critical that the number of items and the number of response options on these items are also constant over time. In general, we should expect the emission matrices to differ in cases where different items are administered over time.

After deciding upon whether the constant emission matrix assumption is feasible researchers then move to the second layer of flowchart to (2a) if the assumption is feasible or (2b) if the emission matrix is likely to vary over time. In the case of a constant emission matrix, we direct researchers to results in previously published papers. For instance, Allman et al. (2009) and Bonhomme et al. (2016) provide results for conventional HMMs that deploy an unstructured emission matrix and Y. Liu et al. (2023) discuss conditions for identifying parameters of RHMMs. One important detail to recognize is that although Allman et al. (2009) and Bonhomme et al. (2016) describe a conventional HMM with one item per time period, their results can be applied to an emission matrix formed as a Khatri–Rao product of item emission matrices (e.g., see Definition 5).

If the answer to the constant emission matrix question at the first decision point is “no” the flowchart proceeds to question (2b), which deals with whether the Markov chain that governs transitions among hidden states is irreducible. As noted above, an irreducible Markov chain is one where it is possible to reach any state from every other state in a finite number of moves. In other words, this decision point forces researchers to grapple with the way they anticipate their phenomenon of interest to change over time. A Markov chain is irreducible if there are no absorbing states, so, researchers interested in designing assessments to study learning would likely answer “no” and continue to (3b). In other cases, researchers may expect respondents to move freely among states and it would be appropriate in these settings to proceed along the “yes” path of the flowchart to (3a).

Decision point (3a) is focused on whether the irreducible Markov chain is coupled with structure in the emission probabilities. If structure is present, the emission probabilities can be modeled as an RHMM and researchers could use binary, polytomous, or nominal restricted models for the emission response probabilities. Figure 2 indicates that the appropriate identifiability results to guide assessment design in this case are stated in Corollaries 1 and 2 and Remark 8. More specifically, RHMMs for binary data require two conditions on the emission probabilities and latent structure. First, there must be at least one pair of adjacent time points with parallel items to ensure the emission matrices are equal (e.g., condition (3) of Corollary 1 and condition (2) of Corollary 2). Second, the latent structure as described by 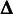 must also satisfy conditions to ensure the minimum number of full rank emission matrices. Strict identifiability requires simple structure items as described in Assumption (2a) whereas generic identifiability requires the weaker condition presented in Assumption (3a).

If the answer of (3a) is “no,” the model is a heterogeneous HMM with time-varying emission matrices. Accordingly, Theorems 1 and 2 present the appropriate conditions for designing identifiable assessment designs. Specifically, researchers that deploy unrestricted emission matrices need to administer one pair of parallel items and care is needed to ensure at least two emission matrices are full rank. One way to satisfy the parallel items assumption is to administer the same item on two adjacent time periods. Theorem 2 shows that heterogeneous HMMs are almost surely identified if the number of response options of the items over time satisfy certain inequalities (e.g., see condition (b)). For instance, the parallel item should be chosen so that the number of response options exceeds the number of hidden states. Furthermore, the product of the number of item response options administered prior to and after the parallel item must also exceed the number of classes.

Decision point (3b) focuses on whether multiple items are administered over time for the reducible Markov chain. The flowchart in Figure 2 shows that there are currently no known identifiability conditions for the case when the answer to (3b) is “no” and only a single item is administered over time.

If multiple items are administered, the flowchart directs us to our last decision point, which is whether there is structure in the response patterns of the multiple items. An answer of “no” implies the emission probabilities are unstructured, so that Theorem 3 includes the appropriate conditions for identifying the model parameters. An important feature of the conditions for the reducible Markov chain case is that conditions must be imposed upon emission probabilities from the first two time points as well as the initial distribution 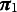 . In particular, the emission matrix from the first time point must consist of two booklets of items, say 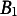 and 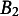 . In the case of heterogeneous HMMs, the model parameters will be strictly identified if the hidden states have distinct response probabilities in the emission matrix formed by 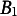 and the emission matrix constructed by 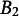 is full rank. Another requirement is that booklet 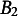 must also be administered at the second time point in order to identify the transition matrix. Also, the initial distribution 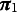 must be strictly positive. This means that practitioners need to collect a representative sample from the population so that respondents are drawn from every hidden state. In the educational context, the assumption that 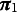 is strictly positive corresponds with ensuring the study consists of students with all types of skill profiles.

Finally, given the case at decision point (4), if the items include structure in their response probabilities the model is an RHMM and Corollaries 3 and 4 and Remark 8 provide guidelines for designing an identifiable assessment. The primary distinction between this case and the results discussed in Theorem 3 is that the Kruskal rank conditions for the emission matrices are replaced with conditions on the 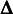 ’s. Satisfying the conditions in Assumption 2 guarantees strict identifiability for binary response RHMMs whereas the conditions in Assumption 3 are needed to generically, or almost surely, identify the binary RHMM parameters. Remark 8 discusses how previous research on the identifiability of more general nominal models can be integrated into our framework to understand the required conditions for the 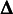 ’s.

## 5 Methods and empirical analyses

This section discusses issues related to the estimation and application heterogeneous HMMs. The first subsection presents a Bayesian model and full conditional distributions for a Gibbs sampling algorithm. The second subsection presents results from a simulation study to demonstrate that satisfying the identifiability conditions produces a consistent estimator. The final subsection presents an application with measures of positive and negative affect.

### 5.1 Bayesian model for heterogeneous HMM

This subsection discusses a Bayesian model for the multi-item, heterogeneous HMM. Let 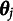 denote the 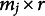 emission matrix for item 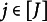 with 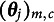 denoting the probability of a response of *m* for members of latent state *c*. We let 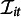 denote the set of items administered at time *j* and 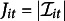 the number of administered items. The conditional likelihood function for the response of individual *i* to item *j* at time *t* is a categorical distribution such that 
(11)

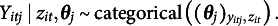

We assume the random variables within the 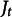 -vector of responses for individual *i* at time *t*, 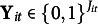 , are conditionally independent given 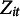 . Accordingly, the likelihood of the responses for individual *i* at time *t* given the value of the hidden state and item parameters is 
(12)

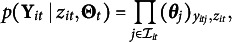

where 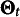 denotes the emission matrices for the administered items at time *t*. We consider independent categorical prior distributions for the hidden states. Specifically, the prior for respondent *i* is: 
(13)

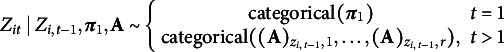

where 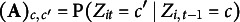 . We consider independent Dirichlet priors for the initial distribution, the columns of the item emission matrices, and the rows of transition matrices as 
(14)

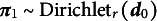


(15)

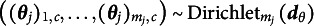


(16)

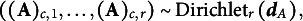

where 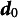 , 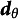 , and 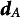 are constants and 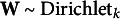 with density function 
(17)

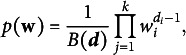

where 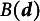 denotes the multivariate beta function.

We deploy a Gibbs sampling algorithm to approximate the posterior distribution. Specifically, we sequentially sample the hidden states, initial distribution, item emission matrices, and transition matrix from the associated full conditional distributions. The full conditional distribution for the hidden state for individual *i* at time *t* is a categorical distribution, 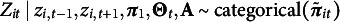 , where element 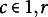 of 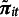 is 
(18)

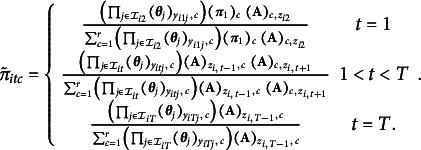



The full conditional distributions for the initial distribution 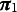 , the columns of the item emission matrices, and the rows of the transition matrix are independent Dirichlet distributions. That is, 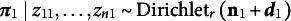 where the *c*th element of 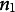 is the number of individuals residing in state *c* at time 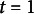 , 
(19)

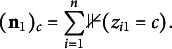

The full conditional conditional distribution for the *c*th column of the emission matrix for item *j* is 
(20)

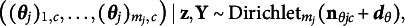

where 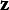 and 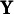 denote the values of the hidden states and observed responses for all individuals across time points and the *k*th element of 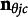 is the number of respondents who reside in state *c* and select option *k* on item *j* over time, 
(21)

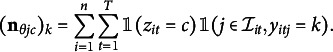

The full conditional distribution for the *c*th row of 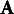 is 
(22)

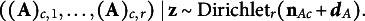

The 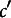 th element of 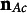 is the number of respondents who transition from state *c* to state 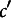 over time, 
(23)

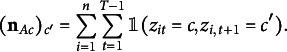



### 5.2 Simulation study

We conducted a simulation study to demonstrate that enforcing the identifiability conditions produces a consistent estimator for the parameters of the heterogeneous HMM. In particular, we focus our attention to the case of the multi-item, heterogeneous HMM. Theorem 3 states that the heterogeneous HMM is identified if: (1) 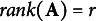 ; (2) 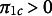 for 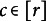 ; and (3) an item administered at time 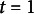 has a full rank emission matrix and is also administered at time 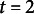 . We simulated data using 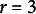 hidden states and 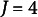 items administered over 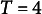 time points. We simulated we responses for items 1 and 2 at time one, item 2 at time 2, item 3 at time three, and item 4 at time four. Item 2 is administered at times 1 and 2 in order to satisfy condition (3) of Theorem 3. A full column rank emission matrix was specified for the first item as 
(24)

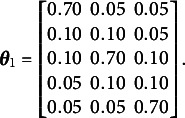

We specified distinct emission matrices for items 2 through 4 by permuting the columns of 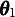 . That is, 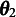 was constructed as columns (3,2,1), 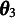 as columns (1,3,2), and 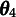 as columns (2,3,1). The data generating value for the transition matrix was 
(25)

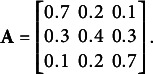

The data generating value for the initial distribution was 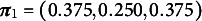 . The data generating transition matrix is full rank and the initial distribution has strictly positive elements, which implies that the data generating parameters satisfy conditions (1) and (2) of Theorem 3.

We simulated data for six conditions with samples sizes of 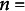 250, 500, 1,000, 2,000, 4,000, and 8,000. We replicated each condition 500 times and computed the mean square error (MSE) for the elements of the four emission matrices, the transition matrix, and the initial distribution. We found evidence using the Gelman-Rubin Rhat statistics of acceptable convergence using a chain length of 5,000 and a burn-in of 1,000.

**Figure 3:**
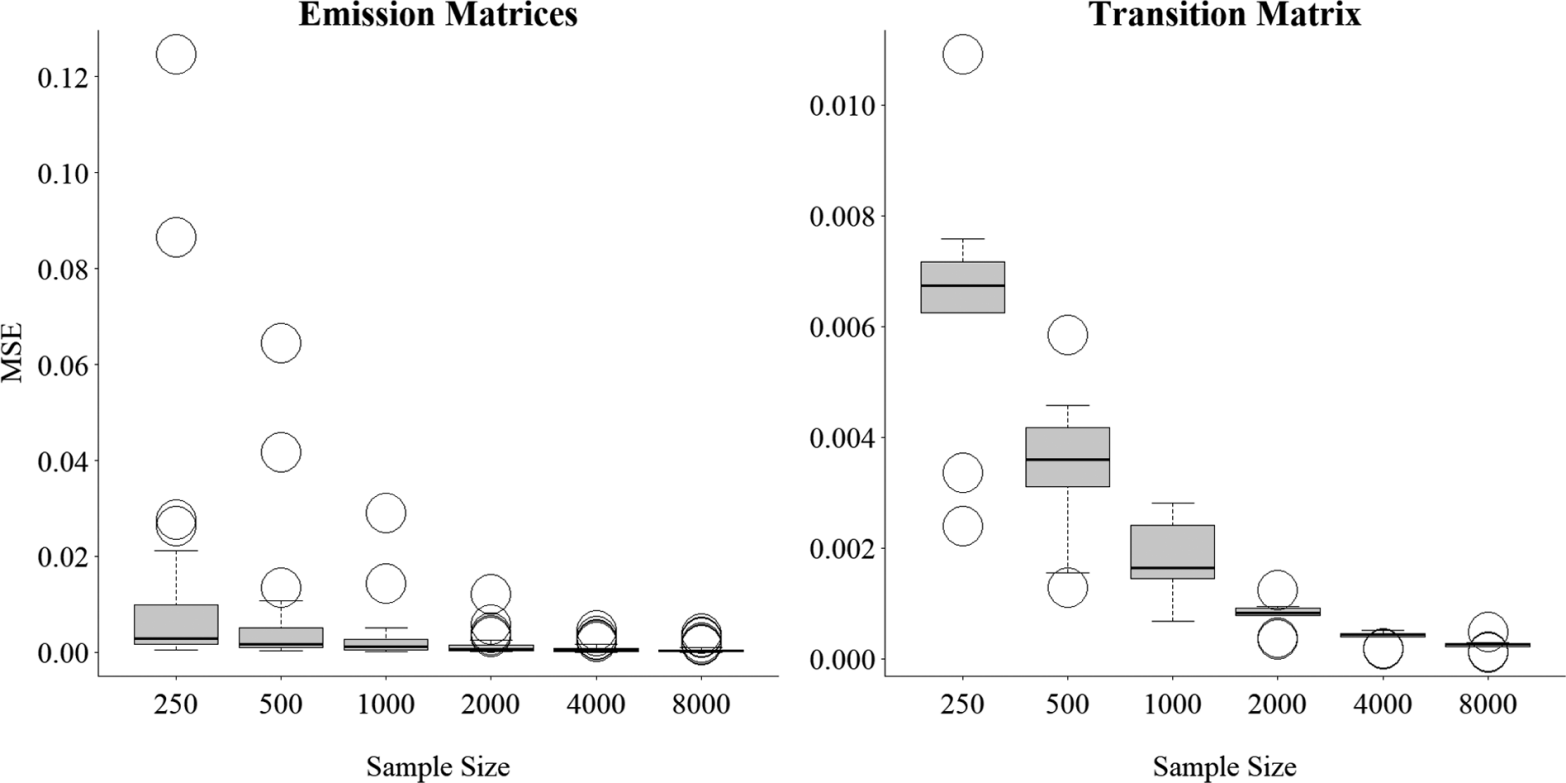
Boxplots of simulated MSE for elements of the emission and transition matrices by sample size.

Table 1 and Figure 3 show that the parameter MSE decreases as the sample size increases. Table 1 reports the average MSE for the elements of 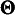 , 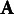 , and 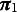 . The average MSE for the elements of the emission matrices equaled 0.0091 for a sample size of 250 and declined with sample size to the value of 0.0005 for 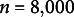 . The average MSE for the elements of 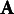 and 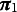 demonstrate a similar pattern. Figure 3 provides more detailed information by presenting boxplots of the MSEs of the 60 elements of the item emission matrices and the nine element of the transition matrix by sample size. Figure 3 shows evidence of consistency in the estimation of 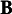 and 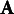 given that the MSEs decline for all elements as the sample size increases.

**Table 1:** Average MSE of the elements of 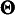 , 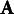 , and 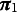 where MSE was computed from 500 replications

*n*	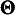	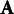	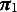
250	0.0091	0.0065	0.0029
500	0.0047	0.0035	0.0021
1,000	0.0023	0.0017	0.0014
2,000	0.0012	0.0008	0.0006
4,000	0.0007	0.0004	0.0003
8,000	0.0005	0.0003	0.0002

### 5.3 Application

Researchers are increasingly interested in developing tools to understand the dynamics of positive and negative affect, mood, and depression (Loossens et al., 2021) and recent research deployed HMMs to study changes in mood and depression (Jiang et al., 2022; Mildiner Moraga et al., 2024). Accordingly, we consider an application involving a dataset of the ten-item Positive and Negative Affect Schedule (PANAS; Shui et al., 2021). The dataset includes 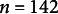 respondents who were surveyed six times a day for five consecutive days for a maximum of 30 observations over time. Participants reported ratings on a five-point scale with anchor endpoints for the value of “1” as “not at all” to a value of “5” to indicate “extremely” for the following item stems: upset, hostile, alert, ashamed, inspired, nervous, determined, attentive, afraid, and active.

Previous research found evidence that responses to mood measures, such as the PANAS, are subject to time-of-day effects where mood changes systematically over the course of a day (Egloff et al., 1995). One implication could be that the psychometric properties of the PANAS items are a function of the time-of-day. Consequently, applying a homogeneous HMM to the PANAS response data may be too restrictive. It is therefore justified to evaluate the relative fit of a homogeneous and heterogeneous HMM to determine whether the emission matrix changes with the time of day.

An important contribution of our article is that our new identifiability theory provides the necessary insight for evaluating the extent to which emission matrices differ within a day. That is, we provide researchers with identifiability conditions for comparing of two identified versions of the homogeneous and heterogeneous HMMs. Therefore our application to the PANAS dataset compares the relative fit of a homogeneous and heterogeneous HMM.

There were instances in the PANAS data where respondents missed one of the six daily data collections. A complete dataset would include 4,260 person by time-of-day by day responses (i.e., 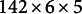 ). The PANAS dataset included 3,789 responses, which implies that participants missed a total of 471 data collections (i.e., 11.1% of the total observations). For the purposes of demonstration, we treat the missing response data as missing at random and impute the hidden state for the missing observations within the Gibbs sampling algorithm. Specifically, the hidden states for the missing observations is imputed by sampling 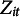 from Equation 18 by replacing the likelihood portion of the probability with the value of one.

We deployed the heterogeneous HMM with 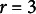 by specifying a distinct emission matrix for the time-of-day (i.e., there were six different emission matrices). We used a Jeffreys’ prior for the columns of the item emission matrices and the rows of the transition matrix. Specifically, the columns of the item emission matrices were distributed as 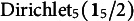 where 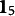 is a vector of five ones and the rows of the transition matrix as 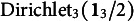 . We generated starting values for the item emission matrices and initial distribution 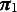 by applying the K-means algorithm to the 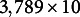 matrix of observed items responses.

We found evidence of convergence using the Gelman-Rubin Rhat statistics by approximating the posterior distribution for both the homogeneous and heterogeneous HMMs by discarding the first 10,000 of 50,000 draws as burn-in. We computed the WAIC (Watanabe & Opper, 2010) for both models in order to evaluate relative fit of the homogeneous and heterogeneous HMMs. The WAIC was smallest for the homogeneous HMM with a value of 85813 versus 86301, which supports the conclusion that PANAS item-level emission matrices were constant over the course of the day.

**Figure 4:**
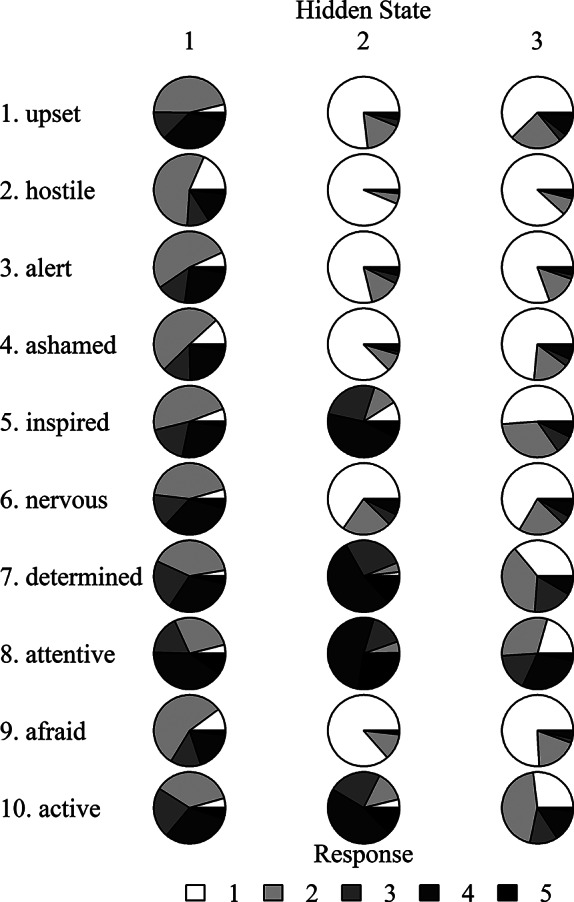
Pie charts of item by class emission probabilities for the PANAS dataset. *Note*: The anchor labels were 1 = “not at all” and 5 = “extremely.”

Figure 4 presents posterior means of the item emission matrices as pie charts of the response probabilities for the ten items and three hidden states. Figure 4 provides evidence that members of state one report more extreme responses to every item. In contrast, the results in Figure 4 suggest that respondents within state two were more optimistic and tended to report lower levels on negative affect items. Additionally, members of state two were more likely to report being inspired, determined, attentive, and active. Finally, members in state three are similar to those in state two in terms of reporting less negative affect. However, members of state three reported lower levels of characteristics such as inspired, determined, and attentive than members of state two.

The posterior mean for the transition matrix was 
(26)

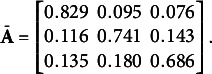

The estimated transition matrix provides evidence that respondents were more likely to remain in their current state. For instance, respondents within the first state had an 82.9% chance of remaining in state one and only had an 9.5% and 7.6% chance of transitioning to states two and three, respectively. In contrast, members of the optimistic state two had a 74.1% chance to remain optimistic, an 11.6% chance to transition into state one, and a 14.3% chance to transition into state three. Finally, members of state three had a 68.6% chance to remain in state three, a 13.5% chance to transition into state one, and an 18.0% chance to transition into the more optimistic state two.

## 6 Discussion

We presented new theory for designing assessments within the HMM and RHMM frameworks. Our results provide practitioners with new insights for creating assessment systems for monitoring student learning and changes in mental health. We extended existing identifiability theory by relaxing the constant emission matrix and irreducibility assumptions. Our results accordingly provide practitioners with guidelines for designing more flexible assessments that are grounded in assumptions that are more likely met in applied settings.

There are several directions for future research. First, we noted in the flowchart in Figure 2 that there are no known identifiability conditions for the time-varying emission matrix case when the Markov chain is reducible and a single item is administered over time. The challenge with this setting is the need to discover a technique to establish identifiability without using the forward projection properties in Lemma 2 of Appendix A derived from the stationarity assumption.

Second, we proposed a Bayesian approach for approximating parameters of the heterogeneous HMM and there are opportunities to extend the algorithm to the case of heterogeneous RHMMs. That is, researchers could consider using Bayesian variable selection methods to impose structure on the emission matrix (e.g., see Balamuta & Culpepper, 2022; Chen et al., 2020; Culpepper & Balamuta, 2023; Jimenez et al., 2023). We established identifiability conditions up to label-switching. One potential challenge with using the Bayesian framework to estimate model parameters is that MCMC algorithms might experience within-chain label-switching, which would impact parameter estimates based on posterior means. Although we did not find evidence of within-chain label-switching in our simulation study or application, within-chain label-switching should be carefully investigated. In cases where within-chain label-switching occurs, researchers should use or modify existing relabeling algorithms (e.g., see Chung, 2019; Erosheva & Curtis, 2017) to permute samples from the posterior distribution.

Third, researchers are also interested in settings where there are attribute hierarchies (e.g., see Chen & Wang, 2023; Tu et al., 2019). In the educational context, attribute hierarchies arise in cases where some attributes can only be mastered after mastery of others. For instance, suppose 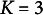 , learning is an absorbing state, and the attributes follow a linear hierarchy where students must first learn attribute 1, then attribute 2, and finally attribute 3. The flowchart in Figure 2 indicates that Corollaries 3 and 4 are the relevant conditions for this setting and a requirement is that the initial distribution 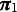 is strictly positive. However, in the case of a linear attribute structure we would expect some elements of 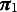 to be zero. For instance, probabilities involving mastery of the second and third attribute without mastery of the first would be zero (e.g., 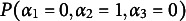 , 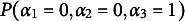 , and 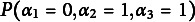 ) in addition to probabilities such as 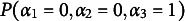 and 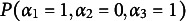 . Our identifiability results can be extended to allow for attribute hierarchies. For instance, Gu & Xu (2023) studied identifiability of restricted latent class models in the presence of attribute hierarchies and their results could be incorporated to establish identifiability conditions for RHMMs when attribute hierarchies exist.

In conclusion, diagnostic models provide researchers with powerful tools for tracking changes in attributes. The increasing availability of longitudinal data will provide researchers the opportunity to leverage information to infer interventions that enhance outcomes. The results we shared in this article will provide researchers with the tools to harness the wealth information available in modern studies.

## Appendix

### A Proof of Theorem 1

The goal of this appendix is to prove Theorem 1. To establish the result we need to understand properties of emission and transition matrices. The first subsection reviews several definitions and results pertaining to emission and transition matrices. Furthermore, we write the heterogeneous HMM as a three-way array using properties of forward- and backward-projection rules. The second subsection discusses backward and forward projections for homogeneous HMMs and then extends these results to the case of heterogeneous emission matrices. Once we are able to write the heterogeneous HMM as a three-way array we use Kruskal’s theorem for the uniqueness of three-way arrays to complete the proof. Accordingly, the third shows how we can write the heterogeneous HMM as a three-way array and applies Kruskal’s theorem.

#### A.1 Properties of emission and transition matrices

We start with by introducing some basic terminology for emission and transition matrices. Definition 6 (Stochastic matrices). A  matrix  with non-negative entries is column-wise stochastic if  and row-wise stochastic if  where for  ,  is an *n*-vector of ones.
Remark 9. The emission matrix  is a column-wise  stochastic matrix and the transition matrix  is a row-wise  stochastic matrix.
Lemma 1 (Properties of irreducible, aperiodic stochastic matrices). If  is an  transition matrix for an irreducible and aperiodic Markov chain then  for  .
Proof. See Stirzaker (2003, p. 416).

#### A.2 Forward and backward projections

We follow previous strategies by writing the HMM as a special case of a mixture model using forward and backward projection rules. Lemma 2 (Bonhomme et al., 2016). Let  be a random response conditioned on  with probabilities specified in the  emission matrix  and stationary distribution,  . Then, the first-order Markovian assumption implies that the emission matrix for:

1. given  is  (backward projection); and
2. given  is  where  (forward projection).

Remark 10. Note that  arises in the distribution for  given  because  requires the conditional distribution of  given  . Consequently,  is the matrix of probabilities governing transitions from  to  .

We next present notation that is essential for constructing the joint distribution of observed responses conditioned upon a common collection of latent states. In particular, our results make use of specialized matrix products, such as the Kronecker and Khatri–Rao products. Definition 7. The Kronecker product two matrices  and  , is denoted by

(A1)

Definition 8. The Khatri–Rao matrix product (i.e., a column-wise Kronecker product) of two matrices  and  , is denoted by

(A2)

We present an example showing how to use the Khatri–Rao product can be used to express the emission matrix for two independent responses. Example 4. Suppose there are two items,  and  such that the responses are conditionally independent given *Z*. The conditional probability for a particular response pattern for  and  is  . We let  denote a  -vector of probabilities for  given  . Also, define  as the  matrix of response probabilities across the *r* classes. Therefore, the conditional independence assumption implies that the  matrix of response probabilities for  and  is  .

We present a two additional examples to demonstrate how to use the forward and backward projection rules and the Khatri–Rao product to write the emission matrix for the responses of two adjacent time points. Example 5. Suppose  and  follow a first-order HMM with emission matrices  and  , respectively, and transition matrix  . Lemma 2. 1 implies that the emission matrix of  given  is  . Note that  and  are conditionally independent given  , so the emission matrix for the combined response patterns for  and  given  is  .
Example 6. Let  and  be the first two observations of a first-order HMM with emission matrices  and  , respectively, and transition matrix  . Lemma 2.2 implies that the emission matrix of  given  is  . The HMM structure implies that  and  are conditionally independent given  , so the emission matrix for the combined response patterns is  .

We will apply the backward and forward projection rules to an arbitrary number of time points, so we introduce notation to represent these emission matrices. Definition 9. Let the backward projected distribution of  given  be defined as  . The distribution for  given  is  .
Definition 10. The forward projected distribution of  given  is denoted as  and the distribution of  given  is denoted as  .

As demonstrated by Allman et al. and Bonhomme et al., we can generalize the expressions in Examples 5 and 6 and write the emission matrix for several observations from the future or the past conditional on a common time  for  . Example 7. Under the HMM structure, we can apply the backward projection rule to derive the conditional distribution of  given  . For instance, the conditional distribution of  given  is

(A3)

Applying the identity recursively implies that the distribution of  given  is,

(A4)

Similarly, the conditional distribution of  ,  , and  given  is

(A5)

A recursive application of the forward projection rule implies that the distribution of  given  is,

(A6)

The forward and backward projected emission matrices rely upon the Khatri–Rao product. The following Lemma presents identities for Khatri–Rao products that will help us to understand properties of forward and backward project emission matrices. Lemma 3 (Khatri & Rao, 1968). Let  and  are matrices of order  and  , and  and  are matrices of order  and  .

- Mixed product property
  (A7)
- Let all of the column vectors of  corresponding to independent column vectors of  be non-null. Then  . Similarly, if all the column vectors of  corresponding to independent column vectors of  are non-null, then  .

The next proposition establishes several important results about forward and backward projected emission matrices. Proposition 1 (Properties of stochastic matrices). Consider the following properties of stochastic matrices:

1. If  is an  row-wise stochastic matrix that is irreducible and aperiodic then  .
2. Backward and forward projection recursive identities:
  - .
3. and  are column-wise stochastic matrices.

Proof. Observe that (1) is established using the property in Lemma 1 of an irreducible, aperiodic  that  and the fact that  . We obtain (2a) because  and  is conditionally independent of  given  . Similarly, part (2b) stems from  , because  and  are conditionally independent given  . For (3), we note that the elements of  and  are products of non-negative elements, so all that remains to show is that the columns sum to one. We proceed with the recursion in 2a for  . Let  with  , so it is a  -vector of ones. Applying Lemma 3 recursively implies  . Similarly, for  ,  is a  -vector of ones. Recursively applying Lemma 3 with  and  implies that  .

We next introduce collapsing properties of backward and forward projected emission matrices. Definition 11. Let  denote the set of integers also in set  , and let

Example 8. Given  , we have

Proposition 2 (Collapsing property). There exists constant collapsing matrices  and  such that,

1. for  and
  (A8)
2. for  and
  (A9)

Proof. We define these constant matrices recursively. For part (1), we note that the collapsing matrix can be written recursively as  for  , where  , and then  . Applying Lemma 3 and (2a) of Proposition 1 implies the first step of the recursion yields:

(A10)

Iterating forward, we can get

(A11)

Then using (2a) and (3) of Proposition 1, we find

(A12)

The proof for part (2) follows in a similar fashion, but in the opposite order. That is, we let  for  , where  , and then  . The first step in the recursion is,

(A13)

Iterating back we find,

(A14)

#### A.3 Three-way arrays

Allman et al. (2009) proved the identifiability of time homogeneous HMMs using Kruskal’s theorem for the uniqueness of three-way arrays (Kruskal, 1976, 1977). We next define a three-way array. Definition 12 (Three-way array). Let  be a three-way array where each  has *r* columns with  defined as

(A15)

where  is column *l* of  for  .

The next example shows that heterogeneous HMMs with, time-varying emission probabilities can be written more generally as a three-way array. Example 9 (Heterogeneous HMMs as a three-way array). We write the heterogeneous HMM model for  as a three-way array by conditioning on  . As shown by Allman et al. (2009), the HMM conditional independence assumption and the requirement that  for the forward-propogation rule in Lemma 2 implies that,

(A16)

We can write Equation A16 as a three-way array as shown in Definition 12. Let  represent the marginal distribution of  and define  as the emission matrix for  given  ,  is a rescaling of the emission matrix for  given  , and  is the emission matrix for  given  . Accordingly, the three-way array representation for Equation A16 given in Definition 12 is

(A17)

where  is element *l* of the stationary distribution  , and  ,  , and  are *l*-th column of  ,  , and  , respectively.
Definition 13. For a matrix  , the Kruskal rank of  ,  , is the largest number *I* such that every set of *I* rows in  are linearly independent (e.g., see Allman et al., 2009).
Theorem 4 (Kruskal, 1976, 1977). Consider the three-way array in Definition 12. If

then  uniquely determines  ,  , and  up to label-switching and rescaling of the columns.

The proof also requires knowledge about the rank of project of matrices, so we present an existing result in the next Lemma. Lemma 4 (Rank of matrix product). If  and  and  then  .

The following proposition establishes the uniqueness of the three emission matrices and stationary distribution for the heterogeneous HMM in Example 9. Proposition 3. Consider the three-way array representation of the marginal distribution  shown in Example 9. Under Assumption 1,  ,  , and  are uniquely identified up to label-switching.
Proof. In Equation A17, we decompose the marginal distribution of  into three tensors  ,  , and  . According to Proposition 1, we have  ,  , and  . Note that (b) implies  , so Lemma 4 implies that  . Therefore, (c) implies that  . Applying property (b) of Lemma 3 implies that  since all columns in  are nonzero. Given  , we can conclude that  . We note that  . We can repeatedly apply (2b) of Proposition 1 to imply that

Part (b) of Lemma 3 implies  because the columns of  are non-null. Recall (c) implies that  , so  . Recall that  , so Lemma 4 implies  , because  . The fact that  implies  . We can continue sequentially in this fashion to show that  ,  ,•,  , which implies  . Now we have  , then by Theorem 4, tensors  ,  , and  are uniquely identified.
Remark 11. Note that Proposition 3 states that the parameters are identifiable up to label-switching, but not rescaling of the columns as stated in Theorem 4. The reason for this is that  is a column-wise stochastic matrix, so the restriction that the columns sum to one eliminate the possibility of arbitrary rescalings. For instance, let  be a diagonal matrix with positive scalars and  . The requirement that  for a column-wise stochastic matrix implies that  .

Now we are able to complete the proof for Theorem 1. Given Assumption 1, by Proposition 3,  ,  , and  are uniquely identified. Next we need to show that parameters  ,  , and  for all *t* can also be identified, i.e.,  ,  , and  if and only if  for all *t*,  , and  . Notice that  implies  . Pre-multiplying by  on both sides implies that  and then  . Given  ,  follows. According to  of Proposition 2, we have  . Given  and let  we get  , then  holds. Now for all  , we have  , then  implies  for all  . Similarly, applying  of Proposition 2 we have  . Given  ,  ,  , and  , then  follows for all  . Thus, HMM parameters are all identified, this completes the proof.

### B Proof of Theorem 2

We extend the notation for the Khatri–Rao product for two matrices to the case with an arbitrary collection of matrices. Definition 14. The Khatri–Rao matrix product of *n* matrices  for  is denoted by

(A18)

Remark 12. Note the product in Definition 14 produces a  matrix.
Example 10. Suppose there are *m* items,  for  , such that the responses are conditionally independent given *Z*. The conditional probability for a particular response pattern  is  . Therefore, the conditional independence assumption implies that the Khatri–Rao product can be used to form the  matrix of response probabilities for  as

(A19)

Lemma 5 (Allman et al., 2009, Lemma 13). Let  ,  , and . Then for generic  ’s,  .

Using Lemma 5, we can establish our Theorem for the generic identifiability of heterogeneous HMMs.

We first need to show that  ,  , and  from Equation A17 generically satisfy Kruskal’s theorem. For generic choice of  , these  and  reduce to  ,  , and  . The  ’s are stochastic matrices so we can express them as  where  is a diagonal matrix of non-zero scalars defined so that the columns sum to one. Recall that both the rank and Kruskal rank are unaffected by multiplying columns by nonzero scalars, so we can apply Lemma 5 to a Khatri–Rao product of  . Therefore, by applying Lemma 5, we have  , and then Theorem 4 generically holds. Thus by Theorem 4, from the joint distribution of the heterogeneous HMM for generic  ,  ,  and  are uniquely determined up to permutations of hidden states.

By employing a similar proof technique as in Theorem 1, and transition matrix  is generically of rank *r*, we can then unravel  ,  , and  to identify the heterogeneous HMM parameters  ,  , and  .

### C Proof of Corollary 1

The proof follows by recalling Chen et al. (2020) showed that if  and  are of the form  with  then  , so the conditions in Assumption 1 are satisfied and Theorem 1 can be applied, which proves the corollary.

### D Proof of Corollary 2

Chen et al. (2020, Theorem 1) establishes that the condition (1) ensures that the column-rank of  and  are generically  . Also,  is generically full rank, so the conditions for Assumption 1 hold generically and Theorem 1 can be applied, which completes the proof.

### E Proof of Theorem 3

The proof follows closely to the proof of Theorem 1 with an exception being the condition that  . First, note that  and  imply that  . Consequently,

(A20)

so the  ,  , and  defined in Equation 10 are unique. The uniqueness of  implies that  is uniquely determined. Pre-multiplying  by  implies that  is unique, so that  is also uniquely determined. The uniqueness of  directly implies  is identified. As described in the proof of Theorem 1, the uniqueness of  and  together imply that terms making up  can be unraveled so that  and  are identified.

### F Proof of Corollary 3

The proof follows by recalling Chen et al. (2020) showed that if  with  and  satisfies Assumption (2b) then  and  , respectively. The conditions of Theorem 3 are satisfied, which proves the corollary.

### G Proof of Corollary 4

Chen et al. (2020, Theorem 1) establishes that the condition (1) ensures that the column-rank of  is generically full rank and  has a Kruskal rank of at least two. Also,  is generically full rank, so the conditions in Theorem 3 generically hold, which completes the proof.
